# List of Diseases at the Bath City Dispensary, from December 1, 1801, to January 1, 1802

**Published:** 1802-02

**Authors:** 


					Diseases in the Bath City Dispensary f 1S9
Jjist of Diseases at the Bath City Dispensary} from December I,
1801, to January i, 1802.
Zinging t t ?
Afthma - - - -
Anafarca - - - - -
Bilious Complaints t - -
Bloody Urine - - - - *
Contufion - - - - - 3
Catarrh - - - - -
Cholic
Chlorofis ?
Eryfipelas- - - - - ?
Excrefcence - * r
Febris Simplex - - >
piftula irj Perinxo 7 - ?
Gravel
Gaftrodynia - - - -
Hernia Humoralis - ?
Hernia - - - - r ?
Leucorrhcea - - - - ?
Lepra ------ !
Nephritic Complaint - ?
Qphthalmia Tarfi - -
Peripneumonia Notha - - 7
Pulmonary Complaints - - 4
Phthifis ------ 6
Phlegmone ----- 1
Pertuffis ------ 2
Podagra ------ j
Phyfconia - - - - - 2
Pfora ------- (
Quotidian - - - - - t
Rheurqatifmus Acqtus - - 9
Synochus Biliofa - - - 7
- - ??? Gaftritica - . - *
Cholerica - - 1
Suppuration - - - - - ?
Siyphilis ^ C
Scrophula - - - - - 2
Strain - ------ I
Tertian ------ 1
T uffis - -- -- -- 5
Ulcerated Legs - - - - 7
Variola Difcreta - - - I
B. In November Sta|ement, inftead of Cjst read Cystorrbcea.
?MEDICAL

				

